# Corrosion Fatigue Damages of Rebars under Loading in Time

**DOI:** 10.3390/ma14123416

**Published:** 2021-06-20

**Authors:** Yaroslav Blikharskyy, Jacek Selejdak, Nadiia Kopiika

**Affiliations:** 1 Department of Highways and Bridges, Lviv Polytechnic National University, 12 St. S. Bandera, 79013 Lviv, Ukraine; Yaroslav.Z.Blikharskyy@lpnu.ua; 2 Faculty of Civil Engineering, Czestochowa University of Technology, 69 St. Dabrowskiego, 42-201 Czestochowa, Poland; 3 Department of Building Constructions and Bridges, Lviv Polytechnic National University, 12 St. S. Bandera, 79013 Lviv, Ukraine; kopijka.nadija.1999@gmail.com

**Keywords:** corrosion fatigue, steel corrosion, cyclic loading, material properties, corrosion in RC constructions

## Abstract

Nowadays, a relatively small number of studies concern the study of corrosion processes in reinforced concrete structures under load. Additionally, rather little research has been carried out concerning changes in the stress–strain state parameters of structures under the simultaneous action of aggressive environment and load. This issue requires additional experimental and theoretical investigation. Determination of mechanical properties, fatigue characteristics and susceptibility to corrosion cracking was performed on samples of reinforcing St3GPF steel. The chemical composition of steel was determined by structural analysis. The spectral method for the determination of alloying elements and impurities in steels is based on the excitation of iron atoms and admixtures by electric discharge, decomposition of radiation into a spectrum, followed by its registration on photoplate with the use of electrograph. Experimental tests of samples in an aggressive environment under the action of statically applied tensile force showed that corrosion damage has little effect on the strength characteristics. At the same time, the decrease in area reduction and the decrease in strain were recorded. Additionally, the action of cyclic loads in an aggressive environment leads to a significant reduction in the fatigue limit to values from 20 to 24% of the yield strength of the original samples, which is 2–3 times lower than the fatigue limit of undamaged samples.

## 1. Introduction and Literature Review

Since reinforcement, such as concrete, is one of the components of reinforced concrete constructions, its corrosion has been studied by many scientists [1,2,3,4,5,6].

The corrosion process could be associated with the deterioration of the metal surface, caused by environmental influences, including oxidation and reduction reactions, which lead to the conversion of metal into oxide, hydroxide or salt. Corrosion mechanisms depend on the great number of external factors, including the following: aggressive environment features, the element stress–strain state, the presence of initial stresses, corrosion spread state, the potential difference, local impairments and damages, uniformity of concrete protective layer and the presence of regions with heterogeneous chemical characteristics. It is important to take into consideration existing defects, cohesion and adhesion properties of the coating, thermal effects and grain size [1,2].

Moreover, other aspects, which have to be definitive in the formation of corrosion parameters, include chemical composition (the water/cement ratio, amount of additions, pH), as well as the diffusion coefficient and the coating thickness [4,5,6].

In some studies, attention is paid to particular environmental conditions and loading effects during the corrosion process. The author of [7] examines such critical parameters as the volume of the protected area, the size effect of the exposed area of the specimen and differential aeration circumstances, which also have a great influence on the level of corrosion damage. Paper [8] considers the effect of delayed chloride exposure on the chloride-induced corrosion initiation. Additionally, such parameters as the concrete type, the effect of aging and their impact on the diffusion coefficient are analyzed. Interesting is the work [9], where on the basis of the profound theoretical research degradation, the law for the flexural strength of corroded PRC beams is suggested.

Studies also analyze the effect of cracks in concrete on the corrosion of reinforcement [10,11,12,13,14]. Experience and research show that where the reinforcement is intersected with cracks formed in the stretched zone of concrete, steel can corrode. Corrosion begins earlier and develops faster, the more aggressive the environment and the greater the width of crack opening is [15]. The degree of corrosion danger for reinforcement in the crack depends on the rebar characteristics such as the cross-section of steel bars and the nature of its corrosion behavior.

In the works [16,17], the rate of rebar corrosion and the intensity (depth) of corrosion damages depending on width of opening of cracks were considered with the use of modern research methods.

Thus, it was found that corrosion of reinforcement occurs in all cracks with an opening over 0.05 mm with the possibility of its amplification, when the opening width increases. By providing structural measures of crack resistance of reinforced concrete structures, it is possible to achieve a crack opening width of 0.05 mm directly before failure [18], which protects the metal from the inside from corrosion.

It is known from practice that the destructive effect of corrosion appears in the weld zones [19]. In [20], the measuring methods of the electrode potentials of the weld zones are considered, which makes it possible to qualitatively assess its corrosion destruction degree depending on the aggressive environment, steel grade and the nature of the stress state. The methods are of practical importance for comprehensive assessment of the durability and reliability of steel structures and can be used to assess the rebar corrosion in reinforced concrete structures.

In the works [21,22], corrosion resistance of reinforcement for different concrete types was considered. One of the ways to solve the problem of durability of structures that are operated in aggressive environments is the use of fiberglass reinforcement [23,24].

In comparison with steel armature, it has a number of advantages: high corrosion resistance, dielectric ability, non-magnetism and radio-transparency. The most effective application of fiberglass reinforcement is for structures made of polymer concrete, polymer silicate concrete and silicate–alkaline acid-resistant concrete. However, such reinforcement has the ability to change their physical and mechanical properties over time.

The tendency for corrosion cracking to occur in steel reinforcement and its dependence on rebar diameter, mechanical properties and magnitude applied of stresses are investigated in the works [25,26,27].

As the result of accelerated corrosion tests, the negative effect on the corrosion cracking susceptibility of thermo-mechanically reinforced reinforcement has been established. Electrochemical methods for assessing the corrosion condition of steel reinforcement of reinforced concrete structures in operating conditions have been developed [28], methods for finding stray voltage have been proposed, and an assessment of electro-corrosion danger has been given.

A significant effect of stress concentrators on steel corrosion has been established. The general trend of the influence of various defects on the strength of metals is widely studied experimentally and theoretically only for geometrically correct stress concentrators [29,30,31].

For damages of irregular shape, such as corrosion ulcers, much less data are available; therefore, it is necessary to experimentally find their effect on the metal mechanical properties in each case. Theoretical calculations in this case are complicated due to the difficult to predict and complex form of surface defects. The decrease in the stress concentrators’ influence on the endurance of steels in corrosive environments can also be caused by the influence of scale factor, which is represented by an increase in corrosion fatigue strength with a decrease in the length of the sample effective part [32,33]. Susceptibility to stress concentration during corrosion fatigue decreases with an increase in the sample diameter; thus, the phenomenon opposite to air tests is observed.

In chloride-containing media, the change in the cut depth in the range of 0.2–8 mm with a 20 mm diameter of the effective cross-section has little effect on the limit of corrosion fatigue. It also does not change significantly with a decrease in the curvature radius of the cut from 15 to 0.1 mm. That is, the corrosive environment eliminates the negative effect of mechanical stress concentration at the fatigue of carbon and low-alloy steels [34,35].

Therefore, when calculating the service life of metal products and reinforcement of reinforced concrete structures, it is important to take into account the possibility of corrosion damage over time. They are effective stress concentrators that cause nonuniformity of the metal stress state, localize plastic deformation in the small zone and can significantly reduce the performance of various structures, constructions, equipment, etc., and require careful study. Therefore, the aim of the work is to study the corrosion fatigue of the reinforcement. It is especially important to investigate this issue in the conditions of the real work of rebars’ corrosion under loading in time.

## 2. Materials and Methods

**Figure 1:**
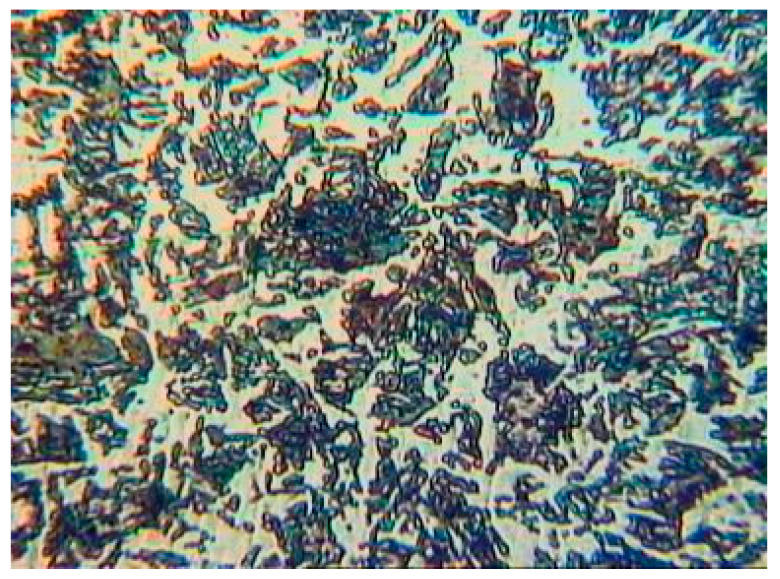
The initial microstructure of reinforcing steel St3GPF.

Investigations of rebar corrosion in aggressive environments under the load action were performed on specially made samples using special equipment. Determination of mechanical properties, fatigue and corrosion fatigue characteristics and corrosion cracking susceptibility was performed on samples of reinforcing steel St3GPF, the chemical composition of which is given in Table 1 and the initial microstructure in Figure 1.

The chemical composition of steel was determined by structural analysis. The spectral method for determining alloying elements and admixtures in steels is based on the excitation of iron atoms and admixtures by electric discharge, the decomposition of radiation into a spectrum, followed by its registration on photoplate using an electrograph. The mass fate of alloying elements and admixtures was determined according to calibration graphs composed for each element using the measured values of the blackness difference of the analytical lines and “internal standards” lines in the spectrograms of samples. In order to compose the calibrator graphs, standard samples and the method of “Three Atoms” were used. The metal sample and standard samples were cleaned before analysis. Surface cavities, scratches, cracks and slag inclusions were not allowed. The sample was clamped at the support lower end, and the upper electrode was the carbon rod. The distance between the ends of the electrodes of 1.5–2 mm was set using a template, and the alternating current arc with an ignition power of 4–9 A was generated, which was powered by a generator from the 220 V network. The length of the arc and the position of the optical axis source were controlled by an aperture mounted on the middle lens and a three-lens illumination system. The spectra were photographed with the use of an electrograph. At least two spectrograms were photographed for each sample. The exposure time was chosen depending on the sensitivity of photographic plates, so that the normal optical background density of the continuous spectrum was provided.

The processed photoplate was decoded under a spectro-projector, selecting analytical line pairs and measuring the blackening of analytical lines and “internal standards” lines with use of a microphotometer (LLC PTP "ASMA-Pribor", Svitlovodsk, Ukraine).

Graduation graphs were composed in coordinates Δ*S*–*lgC*, where *C* is the concentration of particular element in standard samples, and Δ*S* is the difference in blackness of analytical lines. The mass fate of elements in the sample were determined according to graduation graphs by the value of *lgC*, composed with the use of two electrographs.

In order to check the suitability of results of two parallel measurements, the following evaluation Equation (1) was used:

(1) $$X_{1}-X_{2}\leq 2.5\cdot S\cdot X=1,$$

where *S* is the relative standard deviation of single reading; *X*_1_, *X*_2_ are the results of two parallel measurements; *X* is the average of two parallel measurements.

In order to determine the chemical composition of steel, the following apparatus was used: spectrograph ISP-30 (OJSC “LOMO”, St. Petersburg, Russia); generator IVS-28 (LLC “MORS”, Troitsk, Russia); spectro-projector SP-2 (LLC “Carl Zeiss”, St. Petersburg, Russia); microdensitometer MD-100 (“Carl Zeiss Jena GmbH”, Jena, Germany).

**Figure 2:**
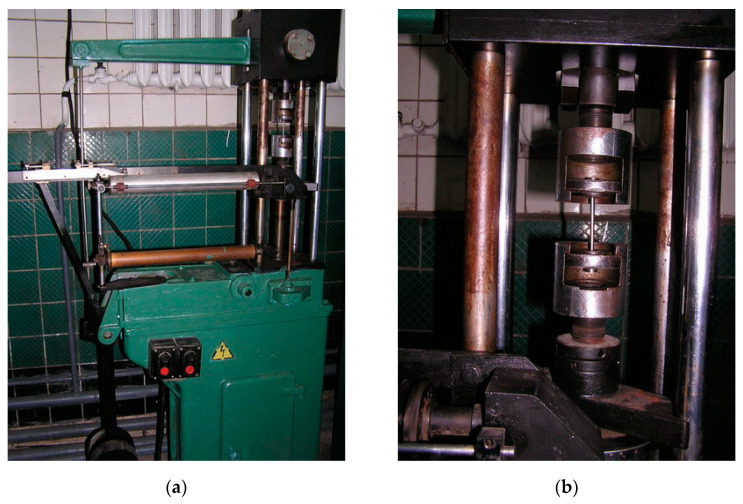
Testing of samples by machine IM-4R: (**a**) general view; (**b**) the sample during the test.

The mechanical properties of reinforcing steel were determined on standard samples with the effective part of 5 mm diameter on the machine IM-4R (JSC “NPO TSNIITMASH”, Moscow, Russia) (Figure 2). Fatigue and corrosion fatigue studies were performed on cylindrical smooth samples with the effective part of 25 mm length and 5 mm diameter. The corrosion cracking susceptibility was determined on samples with effective part of 10 mm diameter. All samples were made of bars with a 20 mm diameter. After turning, the grinding deviation was 0.35 mm. Grinding was performed with EB25SM1K electro-corundum wheels (LLC “Novo Abrasive”, Kharkiv, Ukraine) according to the following mode: the stone linear speed of 30 m/s, the rotation speed of the sample 3 m/s, the grinding depth at the last pass of 0.005 mm/rev.

For determination of the welded joint effect on the corrosion cracking susceptibility of the reinforcing steel to the central part of the sample effective part was welded with 5 mm diameter wire. Welding was performed by the point method on a welding machine with automatic adjustment in factory conditions.

Tests of samples for cyclic durability were performed on a machine IMA-5 (LLC “PO UKRSPETSKOMPLEKT”, Kharkiv, Ukraine) during deformation due to pure bending with rotation at a load frequency of 50 Hz.

**Figure 3:**
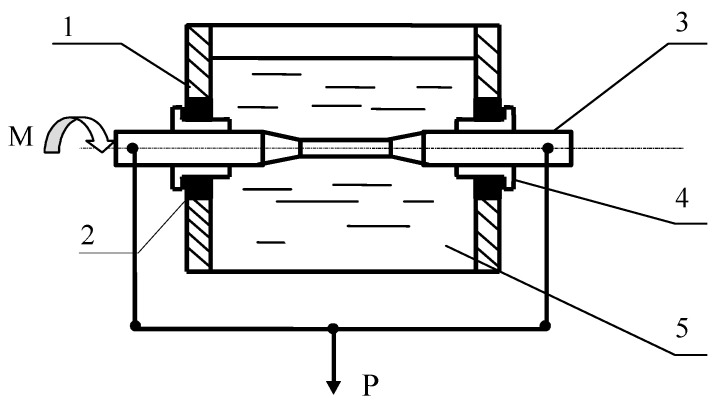
Installation scheme for study of metals in corrosion fatigue conditions. 1: the cell for corrosion environment; 2: oil seal; 3: sample; 4: fluoroplastic plugs; 5: corrosive environment; Р: load.

For corrosion fatigue studies, the sample was placed in a bath for corrosive media. Bath condensation was provided by oil seals and fluoroplastic plugs. The bending moment was transmitted on the rotating sample by the system of levers, to which the force P was applied by gradual of loads selection (Figure 3).

**Figure 4:**
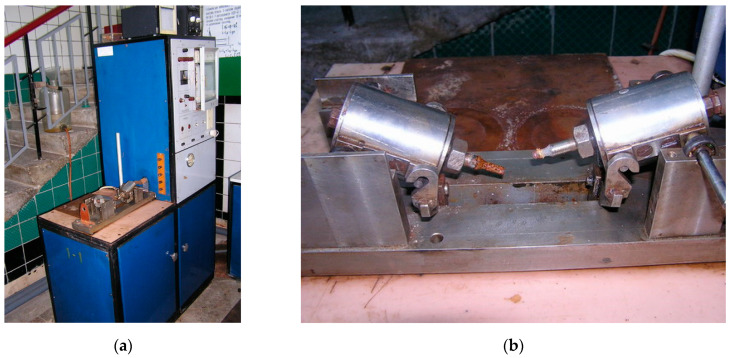
Testing of samples with the use of machine ІМА-5: (**a**) general view; (**b**) the sample after testing.

The general view of test machine IMA-5 is given in Figure 4. Due to bending, the surface layers of the metal samples during rotation undergo the change in stresses from tensile to compressive during one turn of the sample. This ensures the characteristic of cycle asymmetry *ρ* = *σ_min_/σ_max_* = −1.

According to the value of bending moment, the stress value was determined.

The fatigue limit (*σ*_-1с_) was determined on the basis of 10^7^, while the limit corrosion fatigue (*σ*_-1с_) was on the basis of 5 × 10^7^ load cycles, which makes it possible to fully determine the general patterns of environmental influence on the durability of samples with uniform cross-section.

Fatigue curves in the air and media were based on the results of studies of 15 samples for each fatigue curve graph. At stresses corresponding to the fatigue limit and corrosion fatigue limit, at least three samples were tested.

Corrosion damage on the test specimens was obtained by stepwise exposure to a 3% aqueous sodium chloride solution (15 h) and air (8 h) for 15 days or more.

**Figure 5:**
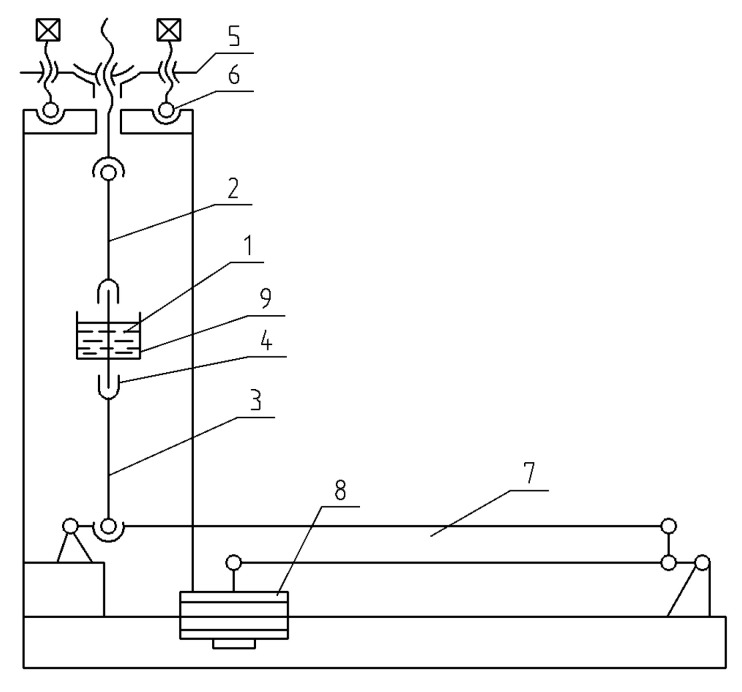
Kinematic scheme of this installation. 1: sample; 2,3: connecting rods; 4: clamps with ball joints; 5: slab; 6: tension screws; 7: levers; 8: loads; 9: container for corrosive environment.

**Figure 6:**
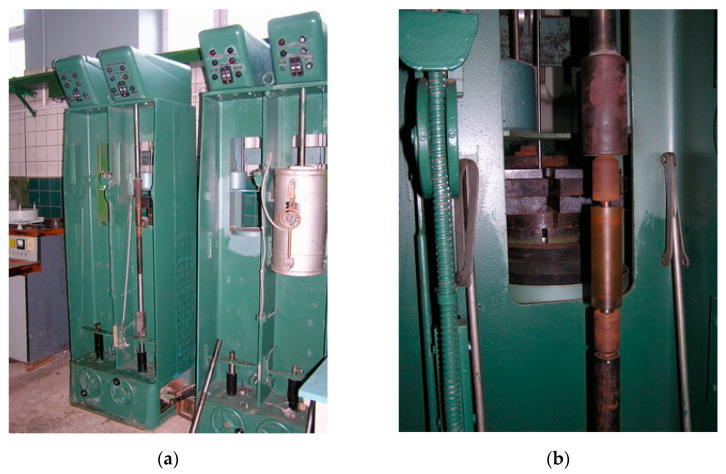
Testing of samples on the installation for cracking research of samples: (**а**) installation general view; (**b**) sample during testing.

For the cracking test, an installation was used in which the stress on the sample was set by constant load. Figure 5 shows the kinematic scheme of this installation, while Figure 6 shows its general view. The corrosion bath was mounted on the sample threaded part. The corrosive environment was changed every 24 h.

**Figure 7:**
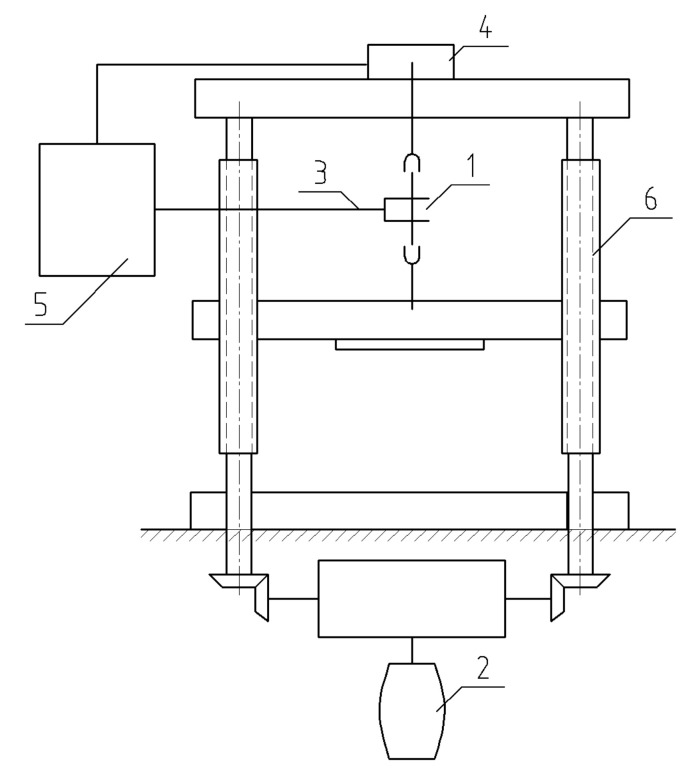
The scheme of testing machine IM-4R on tension. 1: sample; 2: electric motor; 3: strain sensor; 4: load sensor; 5: diagram device; 6: screws.

Uniaxial tensile studies were performed on an IM-4R machine (Figure 7), in which the moving grip is connected to a cargo screw that is connected to a nut. The movement from motor 2 is transmitted to the screw through the reducer. Rotating the screw moves the traverse with grip and, thus, stretches sample 1.

**Figure 8:**
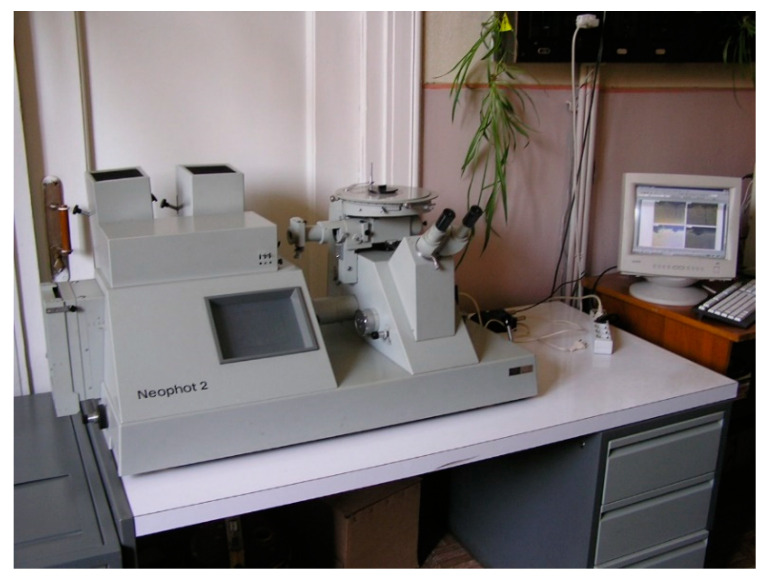
Microscope Neophot-2.

The character of corrosion fatigue damages of samples was studied with the use of microscope МVТ-71 (Urals Optical-Mechanical Plant, Yekaterinburg, Russia) at a magnification ×410 and Neophot-2 (Carl Zeiss Jena GmbH, Jena, Germany) with automatic photo transferring on the calculating machine at a magnification ×250–×500 (Figure 8). For this purpose, samples were previously grinded with different diamond pastes and etched by a solution of 5 mL of НNO_3_ + 95 mL of ethyl spirit.

## 3. Results and Discussion

Testing of initial samples at uniaxial tension has shown (see Table 2) that yield strength and tensile strength for reinforcing steel St3GPF are equal to 318.8 and 510.3 MPa, respectively (the average of four measurements). The relative error does not exceed 1.3%. Corrosion damages, which appear on sample surface over the span of 15–30 days practically do not cause these changes in values. Obtained values are within the experiment error (σ_0.2_ = 314.2–319.0 and σ_u_ = 504.9–510.9 MPa). On the other hand, plastic characteristics (relative elongation (δ) and narrowing (ψ)) change more significantly. For initial samples, the average values were δ = 27.5% and ψ = 62.3% (the relative error does not exceed 3.3%). The value of δ for samples 1 and 2 (the time of exposure was equal to 15 and 20 days) are rather lower than the average value; errors are within the experimental error. For samples 3 and 4 (the time of exposure was equal to 25 and 30 days), the value of δ decreases by 1.04 and 1.22 times, respectively (Table 2). It should be noted that although the exposure time of 15–30 days is relatively small, it provides rather credible results on general deformation and mechanical tendencies. Thus, obtained results (Table 2) provide the clear conception on the qualitative changes in the stress–strain state of studied samples. Namely, in this case, it could be admitted there is a clear tendency towards the reduction in relative elongation values in the presence of corrosion damage on the sample.

As could be seen, the tested samples show lower values of relative narrowing and relative elongation by 4–22% and by 14–22%, respectively.

It should be noted that these changes are especially apparent for the relative narrowing; its values decreased by 1.14–1.22 times for all samples with corrosion damage. These results identify the clear tendency towards a reduction in deformation characteristics and plastic properties of the materials. The increase in time of the exposure of samples in a corrosive environment leads to more significant change in the plasticity characteristics.

Therefore, it is clear that the plasticity characteristics are more sensitive to surface corrosion damage than the strength characteristics. Thus, it could be deduced that tested damaged samples are predisposed to brittle behavior under loading and fragile destruction mechanisms. The understanding of the tendencies, described above, is important in order to reliably assess durability of real constructions, subjected to corrosion influences.

From experimental studies, new properties of reinforcing steel were obtained, which are determined by the deterioration of plastic characteristics during the operation of structures, which could lead to undesirable consequences. Thus, the time from the beginning of rebar plastic operation to the failure is significantly reduced. This phenomenon cannot be allowed, for example, in case of seismic and other dynamic influences. In these cases, some time is required from the beginning of plastic deformation to destruction in order to evacuate people.

**Figure 9:**
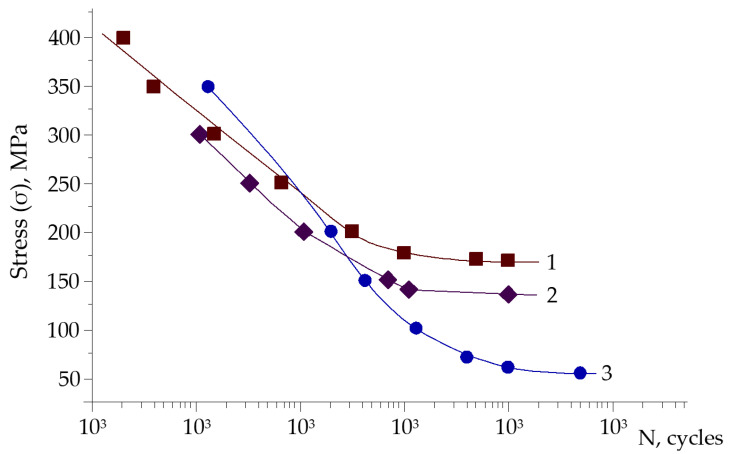
Fatigue curves on the air (1, 2) and in 3% solution of NaCl (3) for steel St3GPF. 1: initial samples without corrosion damages; 2: samples with corrosion damages in the air; 3: samples with corrosion damages in the aggressive environment.

The fatigue study of undamaged rebar exposed to the air has shown that fatigue limit on the basis of 10^7^ cycles is equal to σ_−1_ = 170 MPa (Figure 9, curve 1). Corrosive environment causes this characteristic reduction on the basis of 5 × 10^7^ cycles to σ_-1с_ = 65 MPa (Figure 9, curve 3). Such a sharp decrease in fatigue limit is characteristic of carbon and low-alloy steels.

Durability of samples with corrosion damages (Figure 9, curve 2), which were formed over the course of 15 days, is decreased at 40 MPa (σ_-1_ = 130 MPa), namely by 1.3 times, compared to the undamaged. This difference is about 24% from the fatigue limit of the initial samples. At cyclic stresses of 200 MPa, the durability of damaged samples in comparison with initial samples is ~2.0 times lower.

**Figure 10:**
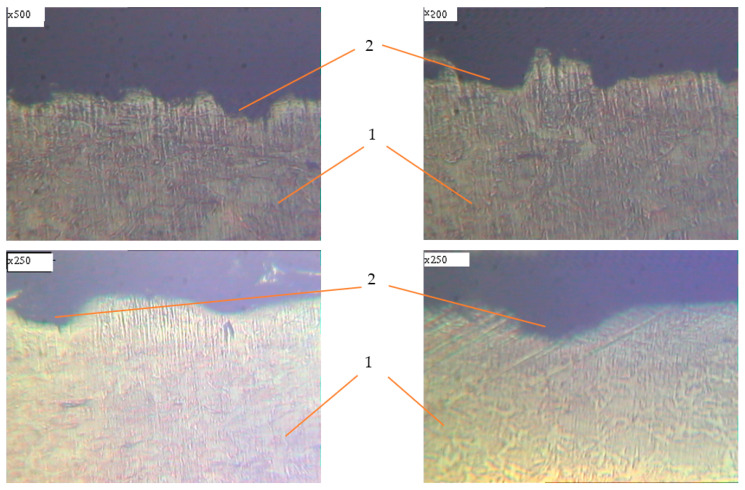
The character of damages for reinforcing steel after 15 days of exposure in corrosive environment. 1: undamaged metal; 2: corrosion ulcer.

**Figure 11:**
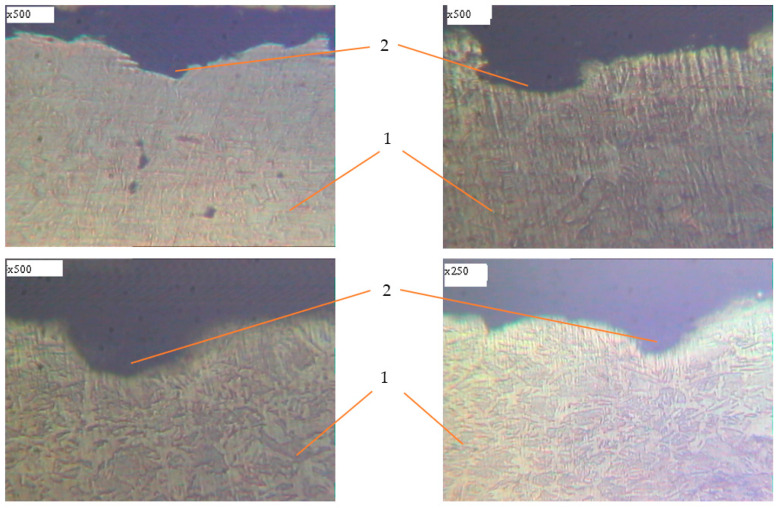
The character of damages of reinforcing steel after 20 days of exposure in corrosive environment. 1: undamaged metal; 2: corrosion ulcer.

**Figure 12:**
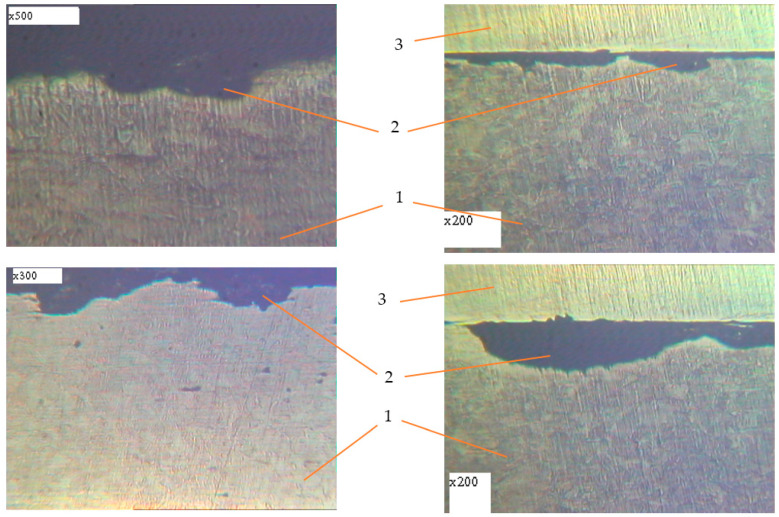
The character of damages of reinforcing steel after 25 days of exposure in corrosive environment. 1: undamaged metal; 2: corrosion ulcer; 3: standard smooth polished metal plate.

**Figure 13:**
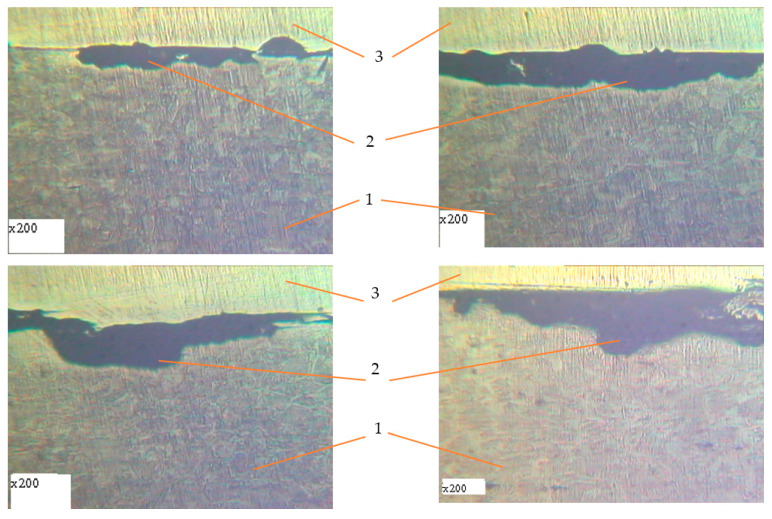
The character of damages of reinforcing steel after 30 days of exposure in corrosive environment. 1: undamaged metal; 2: corrosion ulcer, 3: standard smooth polished metal plate.

**Figure 14:**
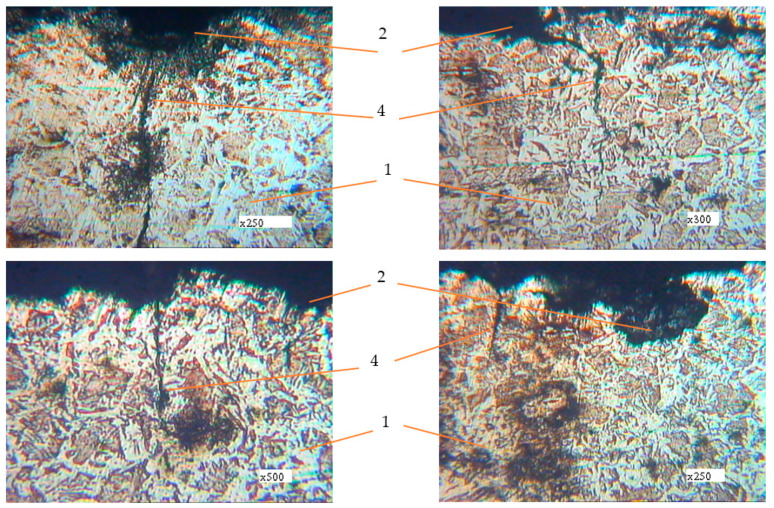
Metallography of samples with corrosion damage (15 days of exposure) after fatigue tests in air (±σ = 170 MPa). 1: undamaged metal; 2: corrosion ulcer; 4: corrosion crack.

**Figure 15:**
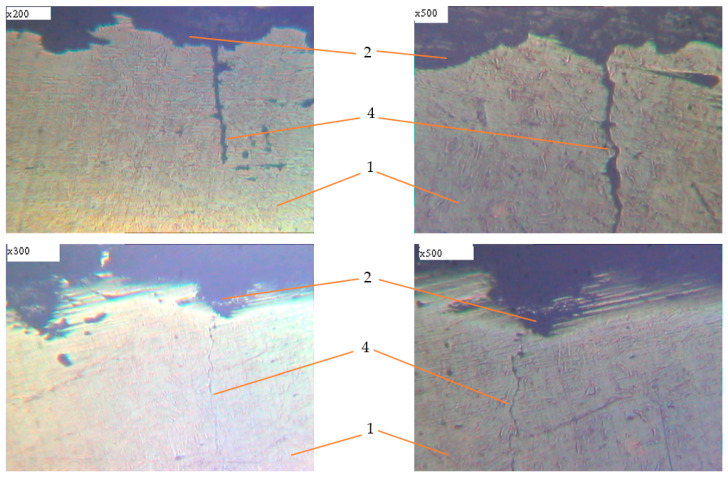
Metallography of samples with corrosion damage (15 days of exposure) after fatigue tests in air (±σ = 200 MPa). 1: undamaged metal; 2: corrosion ulcer; 4: corrosion crack.

**Figure 16:**
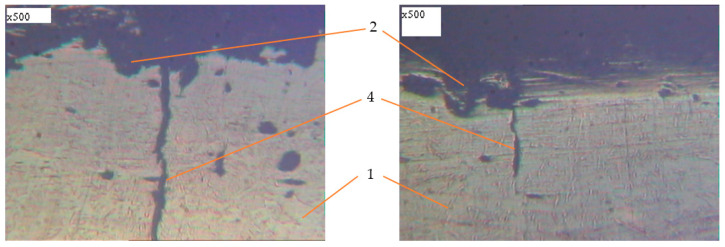
Metallography of samples with corrosion damage (15 days of exposure) after fatigue tests in air (±σ = 250 MPa). 1: undamaged metal; 2: corrosion ulcer; 4: corrosion crack.

Metallographic studies of the surface of sections of the reinforcement samples after the test were performed with the use of an electronic microscope with magnification by 200–500 times. Photos of individual sections are given in Figure 10, Figure 11, Figure 12, Figure 13, Figure 14, Figure 15 and Figure 16. Herewith, individual surfaces of sections were compared with a standard polished metal plate, which made it possible to measure the depth of corrosion damage (Figure 12 and Figure 13).

The analysis of the obtained results shows that in all samples with corrosion damage, which were subjected to an aggressive environment for 15–30 days, corrosion ulcers up to 60–70 μm are formed. In most cases, the form is sharper (Figure 10); therefore, from them, fatigue cracks arise (Figure 14, Figure 15 and Figure 16), especially under the action of cyclic loads. The deepest lesions are mostly the bluntest (Figure 11).

In addition, increasing the exposure time for the samples in an aggressive environment does not significantly increase the depth of damage, but leads to an increase in the damaged area (Figure 12 and Figure 13). Under the action of cyclic loads, fatigue cracks arise in the tops of corrosive ulcers (Figure 14, Figure 15 and Figure 16). The smallest depth of a corrosion ulcer from which fatigue cracks begin is equal to ~15 microns (Figure 15 and Figure 16).

A characteristic feature of the destruction of such samples is that several cracks arise on the surface, one of which becomes dominant and leads to destruction. For smooth samples without corrosion damage, only one fatigue crack always arises and grows, which leads to destruction.

**Figure 17:**
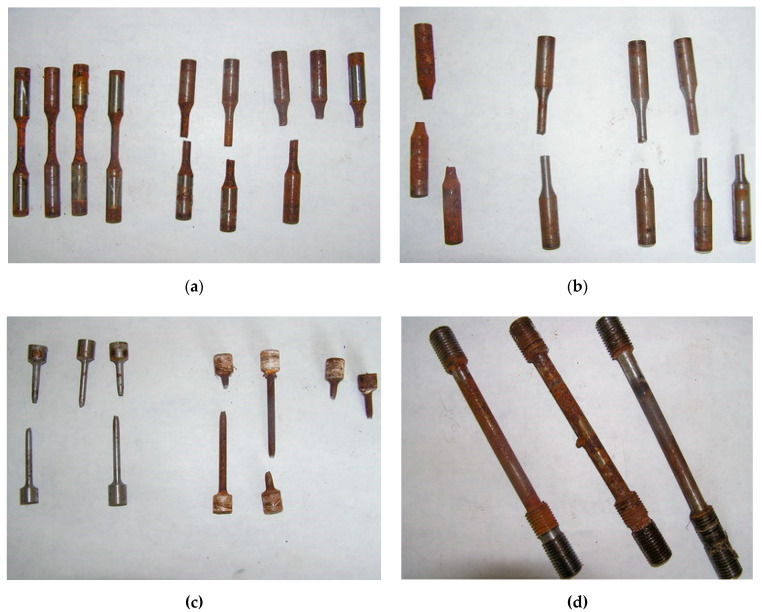
Destruction character for testing rebar samples. (**a**,**b**) Tested samples for corrosion fatigue in aggressive environments; (**c**) tested samples for static load in the air; (**d**) tested samples for corrosion cracking with a welded rebar.

Testing of samples (initial and samples with welded steel bar) under static load (0.9·σ_02_) in 3% NaCl solution over the course of 720 h did not cause their destruction. At loads of 0.9·σu samples of such a type did not collapse over the course of 120 h. Rebar samples and their destruction character are shown on Figure 17.

Therefore, it can be stated that corrosion damage in the form of surface ulcers of different shapes and depths has a greater impact on the plasticity and durability characteristics under cyclic loads than on the strength of reinforcing steel under static load.

## 4. Conclusions

In the study, profound research was carried out concerning changes in the stress–strain state parameters of structures under the simultaneous action of aggressive environment and load.

Experimental tests of reinforcing steel samples in an aggressive environment under the action of statically applied tensile force showed that corrosion damage has little effect on the strength characteristics. At the same time, a decrease in the value of relative narrowing by 4–22% and relative elongation by 14–22% was recorded, which identifies tendency towards changes in deformation properties of the material. It was identified that the plasticity characteristics are more sensitive to surface corrosion damage than the strength characteristics. Thus, it could be deduced that tested damaged samples are predisposed to brittle behavior under loading and fragile destruction mechanisms.

Another important aspect is that under the action of cyclic load in aggressive environment at *ρ* = *σ_min_/σ_max_* = −1, corrosion damages cause a significant decrease in the fatigue limit up to 20–24% from the yield strength of initial rebar samples, which is 2–3 times lower than the fatigue limit of samples without corrosion damages.

From experimental studies, new properties of reinforcing steel were obtained, which are determined by the deterioration of plastic characteristics during the operation of structures, which could lead to undesirable consequences. Thus, the time from the beginning of rebar plastic operation to the failure is significantly reduced, and durability properties of reinforcement are considerably decreased, which should be taken into account in order to reduce the risk of accidental dangerous situations. This aspect could be decisive for structure reliability, especially in the case of seismic and other dynamic influences.

Experimental investigation, introduced in the work, confirmed the necessity of further theoretical and experimental research of this issue. Understanding of the tendencies, described above, is important in order to reliably assess the sustainability of real constructions subjected to corrosion influences.

## Figures and Tables

**Table 1:** Chemical composition of reinforcing steel.

Element	The Element Content, %
C	0.18
Si	0.15
Mn	0.9
Cr	0.1<
Ni	0.1<

**Table 2:** Mechanical properties of the steel St3GPF.

Sample	σ_u_, MPa	σ_0.2_, MPa	ψ, %	δ, %
Initial 1	509.5	319.7	63.5	26.7
Initial 2	504.5	314.7	61.6	28.4
Initial 3	514.5	318.7	62.5	27.2
Initial 4	512.5	322.2	61.8	27.7
Average value	510.3	318.8	62.3	27.5
Sample 1 with CD ^1^ (15 days of exp.)	508.3	315.6	54.6	27.1
Sample 2 with CD ^1^ (20 days of exp.)	510.9	315.9	53.3	26.8
Sample 3 with CD ^1^ (25 days of exp.)	509.5	319.0	53.3	26.4
Sample 4 with CD ^1^ (30 days of exp.)	504.5	314.2	51.0	22.2

^1^ CD: corrosion damages.

## Data Availability

The data presented in this study are available on request from the corresponding author.
